# Population Genetic Polymorphism of Skeletal Muscle Strength Related Genes in Five Ethnic Minorities in North China

**DOI:** 10.3389/fgene.2021.756802

**Published:** 2021-10-11

**Authors:** Bonan Dong, Qiuyan Li, Tingting Zhang, Xiao Liang, Mansha Jia, Yansong Fu, Jing Bai, Songbin Fu

**Affiliations:** ^1^ Laboratory of Medical Genetics, Harbin Medical University, Harbin, China; ^2^ Key Laboratory of Preservation of Human Genetic Resources and Disease Control in China (Harbin Medical University), Ministry of Education, Harbin, China; ^3^ Editorial Department of International Journal of Genetics, Harbin Medical University, Harbin, China; ^4^ Scientific Research Centre, The Second Affiliated Hospital of Harbin Medical University, Harbin, China

**Keywords:** skeletal muscle strength-related genes, SNP, ethnic groups, phylogenetic relationship, population genetics

## Abstract

Musculoskeletal performance is a complex trait influenced by environmental and genetic factors, and it has different manifestations in different populations. Heilongjiang province, located in northern China, is a multi-ethnic region with human cultures dating back to the Paleolithic Age. The Daur, Hezhen, Ewenki, Mongolian and Manchu ethnic groups in Heilongjiang province may have strong physical fitness to a certain extent. Based on the genetic characteristics of significant correlation between some important genes and skeletal muscle function, this study selected 23 SNPs of skeletal muscle strength-related genes and analyzed the distribution of these loci and genetic diversity in the five ethnic groups. Use Haploview (version 4.1) software to calculate the chi-square and the Hardy-Weinberg equilibrium to assess the difference between the two ethnic groups. Use R (version 4.0.2) software to perform principal component analysis of different ethnic groups. Use MEGA (version 7.0) software to construct the phylogenetic tree of different ethnic groups. Use POPGENE (version 1.32) software to calculate the heterozygosity and the F_ST_ values of 23 SNPs. Use Arlequin (version 3.5.2.2) software to analyze molecular variance (AMOVA) among 31 populations. The results showed that there was haplotype diversity of *VDR*, *angiotensin-converting enzyme*, *ACTN3*, *EPO* and *IGF1* genes in the five ethnic groups, and there were genetic differences in the distribution of these genes in the five ethnic groups. Among them, the average gene heterozygosity (AVE_HET) of the 23 SNPs in the five populations was 0.398. The F_ST_ values of the 23 SNPs among the five ethnic groups varied from 0.0011 to 0.0137. According to the principal component analysis, the genetic distance of Daur, Mongolian and Ewenki is relatively close. According to the phylogenetic tree, the five ethnic groups are clustered together with the Asian population. These data will enrich existing genetic information of ethnic minorities.

## Introduction

Skeletal muscle is one of the most dynamic and plastic tissues of the human body, and it is an important part of the human body. The skeletal muscles are involved in various functions of human life. From a mechanical perspective, the main function of skeletal muscle is to convert the body’s chemical energy into mechanical energy, so that the body can generate force and strength, and then generate movement to maintain or benefit human health. From a metabolic perspective, the roles of skeletal muscle include promoting basal energy metabolism, storing important substrates such as amino acids and carbohydrates, and providing most of the oxygen and energy for human movement (Frontera and Ochala, 2015).

With the development of exercise physiology, studies have found that acquired physical training has an important and positive effect on the improvement of human muscle mass, strength and function (Phu et al., 2015). In addition, genetic differences can influence the ability of the body’s skeletal muscles to produce and use energy during exercise (Yan et al., 2016). Studies have highlighted a significant correlation between potentially important genes and musculoskeletal function. For example, the *VDR* gene may have a positive effect on skeletal muscle (Książek et al., 2019), and the *IGF1* gene increases muscle mass and improves skeletal muscle regeneration (Vassilakos and Barton, 2018), and the *EPO* gene promotes differentiation and survival of myoblasts (Lamon and Russell, 2013). In addition, other genes involved in skeletal muscle strength include the endurance gene *ACE* (Ahmetov and Fedotovskaya, 2015), and the strength-related genes, such as *ACTN3* (Ahmetov and Fedotovskaya, 2015; Seto et al., 2021), *AGT* (Pickering et al., 2019), *PPARG* (Ahmetov and Fedotovskaya, 2015; Norouzi et al., 2019) and *IL6* (Pickering et al., 2019). Due to environmental and genetic factors, there are different manifestations in different ethnic groups (Pitsiladis et al., 2016). For instance, the frequencies of the three *ACE* genotypes (II, ID, DD) were 25, 50, and 25%, respectively, in Caucasian populations (Jones et al., 2002), which were not significantly different from those of Asian populations in Korea (23, 66, and 11%, respectively) (Oh, 2007). Other studies have found that the ID genotype is significantly associated with outstanding endurance quality in both European and African American populations (Weyerstraß et al., 2018). The A allele of rs699 locus of *AGT* gene was significantly correlated with Brazilian endurance quality (Guilherme et al., 2018). CT genotype of *ACTN3* gene was markedly correlated with explosive power of Caucasian. CC genotype was substantially correlated with Asian explosive power. The T allele or TT genotype was significantly correlated with the explosive power of both Caucasian and Asian male populations, and the TT genotype also significantly affected the explosive power of Russian athletes (Weyerstraß et al., 2018). CC genotype of *AGT* gene has a high performance in Polish power athletes, with a genotype frequency of 40% (Zarębska et al., 2013). The C allele of *IL6* was positively associated with athletic ability in Israelis of Ethiopian descent, which not only improved speed but also improved training recovery (Ben-Zaken et al., 2021). China is a multi-ethnic country, consisting of the Han nationality and 55 ethnic minorities, of which the population of 55 ethnic minorities accounts for about 8% of the total population. To a certain extent, it provides abundant genetic resources for the study of genes related to skeletal muscle strength. Heilongjiang province, located in northern China, is a multi-ethnic region with human culture since the Paleolithic Age. To some extent, the Daur, Mongolian, Ewenki, Manchu and Hezhen belong to the Altaic language family in Heilongjiang may have stronger physical fitness. According to reports, the grip strength of Mongolian, Daur and Ewenki adults is significantly higher than the national level (Dong et al., 2004). In addition, some scholars believed that some indexes of physical characteristics in Hezhen people are slightly higher than those of Han people due to engaged in fishing and hunting activities for a long time (Chen et al., 1999; Wang et al., 2014). Some scholars sorted out and counted the relevant materials of 263 Manchu college students aged 19 to 22, and found that the physical fitness of Manchu college students was significantly better than that of Han (Bi, 1993).

Single nucleotide polymorphisms (SNPs) refer to DNA sequence polymorphisms caused by a single nucleotide variation at the genome level, with a frequency generally greater than 1% in the population. SNP is closely related to the genetic traits of populations and can be used as genetic markers for the genetic structure of different populations (Galinsky et al., 2019). Based on the genetic characteristics of significant correlation between some important genes and skeletal muscle function, this study intends to select 23 SNPs in *AGT* (rs699, rs4762, rs5051, rs5050), *PPARG* rs3856806, *IL6* rs2066992, *ACE* (rs4309, rs4331, rs4341, rs4343, rs4362), *ACTN3* (rs1815739, rs540874), *EPO* (rs1617640, rs551238), *IGF1* (rs5742714, rs1520220, rs5742612, rs972936), *VDR* (rs7975232, rs757343, rs2228570, rs11568820) genes. We analyzed the allele frequency of these loci in Daur, Hezhen, Ewenki, Mongolian and Manchu, and compared with the 26 populations from 1,000 genome project, to investigate the genetic polymorphism of skeletal muscle strength related genes in the five ethnic groups and to provide theoretical support for explaining the genetic polymorphism of skeletal muscle strength related genes between different populations.

## Materials and Methods

### Study Populations

**FIGURE 1:**
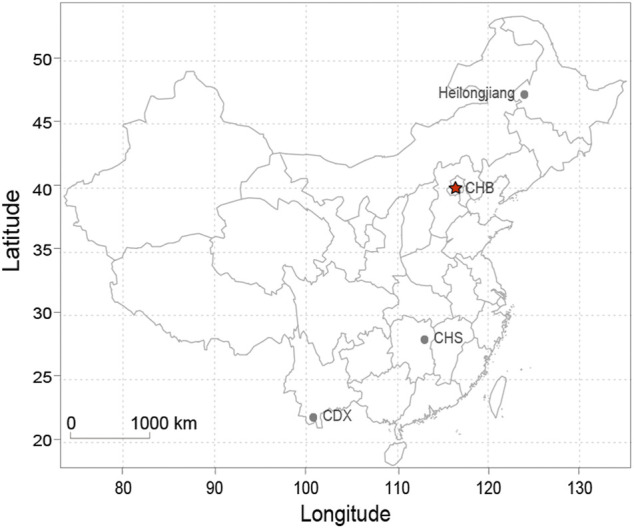
The geographical distribution of eight ethnic groups in China. Note: CHB: Han Chinese in Beijing; CHS: Southern Han Chinese; CDX: Chinese Dai in Xishuangbanna.

Blood samples were collected from 882 unrelated individuals (413 males, 469 females, 45 average age) belonging to five Chinese ethnic minorities in Heilongjiang province at least three generations. These individuals include 233 Daur individuals, 106 Mongolian individuals, 73 Ewenki individuals, 220 Manchu individuals, and 250 Hezhen individuals. The geographical distribution on the map is shown in Figure 1. The study was carried out in strict accordance with the Declaration of Helsinki and approved by the Ethics Committee of the Harbin Medical University. All the participants signed a written informed consent form.

### DNA Extraction and Genotyping

Genomic DNA was extracted from 200 μl blood using the QIAamp DNA Blood Mini Kit (Qiagen, Hilden, Germany) according to the manufacturer’s instructions. Genotyping was performed using the SNPscan™ Kit (Genesky Biotechnologies Inc., Shanghai, China) according to the manufacturer’s instructions.

### Database Data

The genotype and allele frequency data of individuals from the 26 populations in the world were downloaded from the ensemble database at http://grch37.ensembl.org/Homo_sapiens/Tools/DataSlicer The abbreviations and full names of the 26 populations in the world were downloaded from the https://www.ncbi.nlm.nih.gov/variation/tools/1000genomes.

### Statistical Analysis

Chi-square and Hardy-Weinberg equilibrium were calculated to assess the differences between two populations using the Haploview software, the linkage disequilibrium and the haplotype analysis of SNPs also were performed by it (Barrett et al., 2005). In the haplotype analysis the *r* ^2^ threshold was 0.8. Phylogenetic tree was generated using the UPGMA dendrogram method in MEGA7 (Kumar et al., 2016). The parameter such as AVE_HET, FST, Nm and the Nei’s genetic distance based on UPGMA of the five ethnic groups were calculated using the POPGENE software (Yeh et al., 1997). Principal component analysis (PCA) were carried out in the R packages “factoextra” and “ggplot2” (Luu et al., 2017; Singh and Soman, 2019). Analysis of molecular variance (AMOVA) was calculated by Arlequin (Excoffier et al., 2007).

## Results

### Genotyping Data and Hardy-Weinberg Test

The genotype distribution in the study is summarized in Table 1. The 23 SNPs included in the study were all in line with Hardy-Weinberg equilibrium (*p* > 0.05). The minimum allele frequencies and genotype frequencies of 23 SNPs in five populations are summarized in Table 2 and Supplementary Table S1 respectively.

**TABLE 1:** The genotype distribution and Hardy-Weinberg equilibrium test for the 23 SNPs in five ethnic populations from China.

Gene	Loci	A/B	AA^a^	AB^a^	BB^a^	HW*P*val
*AGT*	rs699	G/A	567	271	36	>0.05
	rs4762	G/A	749	113	2	>0.05
	rs5051	T/C	552	265	39	>0.05
	rs5050	T/G	605	229	30	>0.05
*PPARG*	rs3856806	C/T	590	262	21	>0.05
*IL6*	rs2066992	T/G	396	378	108	>0.05
*EPO*	rs1617640	A/C	449	359	72	>0.05
	rs551238	T/G	431	374	77	>0.05
*ACTN3*	rs1815739	C/T	460	253	167	>0.05
	rs540874	G/A	442	233	169	>0.05
*IGF1*	rs5742714	C/G	651	204	21	>0.05
	rs1520220	C/G	415	303	163	>0.05
	rs5742612	A/G	477	345	60	>0.05
	rs972936	C/T	416	332	133	>0.05
*VDR*	rs7975232	C/A	481	345	56	>0.05
	rs757343	C/T	546	240	24	>0.05
	rs2228570	G/A	393	311	164	>0.05
	rs11568820	C/T	410	311	143	>0.05
*ACE*	rs4309	T/C	402	346	132	>0.05
	rs4331	G/A	403	356	123	>0.05
	rs4341	C/G	402	355	124	>0.05
	rs4343	A/G	404	355	122	>0.05
	rs4362	C/T	422	325	132	>0.05

a AA wild homozygote, AB heterozygote, BB mutant homozygote.

**TABLE 2:** The minimum allele frequencies of 23 SNPs in five populations.

Loci	Daur	Mongolian	Ewenki	Manchu	Hezhen
rs699	0.238	0.197	0.205	0.147	0.197
rs4762	0.056	0.069	0.041	0.06	0.093
rs5051	0.239	0.208	0.225	0.156	0.192
rs5050	0.144	0.154	0.16	0.151	0.211
rs3856806	0.178	0.199	0.178	0.172	0.161
rs2066992	0.33	0.429	0.425	0.311	0.3
rs1617640	0.282	0.324	0.342	0.282	0.26
rs551238	0.307	0.321	0.336	0.293	0.278
rs1815739	0.429	0.443	0.452	0.45	0.476
rs540874	0.425	0.441	0.459	0.5	0.478
rs5742714	0.139	0.105	0.116	0.166	0.141
rs1520220	0.367	0.376	0.404	0.425	0.358
rs5742612	0.238	0.259	0.281	0.284	0.266
rs972936	0.367	0.381	0.404	0.436	0.36
rs7975232	0.236	0.33	0.336	0.277	0.212
rs757343	0.133	0.2	0.229	0.215	0.169
rs2228570	0.337	0.44	0.4178	0.435	0.413
rs11568820	0.425	0.368	0.37	0.481	0.338
rs4309	0.38	0.365	0.37	0.336	0.422
rs4331	0.382	0.349	0.384	0.307	0.412
rs4341	0.382	0.348	0.384	0.311	0.412
rs4343	0.382	0.348	0.384	0.311	0.408
rs4362	0.393	0.365	0.39	0.35	0.434

### The Frequencies of the Polymorphisms Among Different Populations

The SNPs with statistical differences in the comparison between the two ethnic groups are summarized in Table 3. In the comparison between Daur and Ewenki, Daur and Hezhen, Daur and Manchu, Daur and Monngolin, there were three, four, eight and four SNPs with statistical difference, respectively. In the comparison between Ewenki and Hezhen, Ewenki and Manchu, there were three and two SNPs with statistical difference, respectively. In the comparison between Manchu and Hezhen, Mongolin and Hezhen, Mongolin and Manchu, there were eleven, two and three SNPs with statistical difference, respectively (*p* < 0.05).

**TABLE 3:** Summary statistical different SNPs after Pairwise comparison of five populations.

Populations	Gene	Loci	Assoc allele	Chi square	*p* Value
Daur vs Ewenki	*IL6*	rs2066992	G	4.318	0.0377
	*VDR*	rs7975232	A	5.731	0.0167
		rs757343	T	7.547	0.006
Daur vs Hezhen	*VDR*	rs2228570	A	5.91	0.0151
		rs11568820	C	7.601	0.0058
	*AGT*	rs4762	A	4.737	0.0295
		rs5050	G	7.459	0.0063
Daur vs Manchu	*ACE*	rs4343	A	4.975	0.0257
	*VDR*	rs757343	T	9.932	0.0016
		rs2228570	A	9.076	0.0026
	*ACE*	rs4331	G	5.653	0.0174
		rs4341	C	4.975	0.0257
	*IGF1*	rs972936	T	4.541	0.0331
	*AGT*	rs699	G	12.014	0.0005
		rs5051	T	9.64	0.0019
Daur vs Mongolian	*IL6*	rs2066992	G	6.16	0.0131
	*VDR*	rs7975232	A	6.622	0.0101
		rs757343	T	4.569	0.0326
		rs2228570	A	6.396	0.0114
Ewenki vs Hezhen	*IL6*	rs2066992	T	7.965	0.0048
	*VDR*	rs7975232	C	9.469	0.0021
	*AGT*	rs4762	A	4.042	0.0444
Ewenki vs Manchu	*IL6*	rs2066992	T	6.274	0.0123
	*VDR*	rs11568820	T	5.256	0.0219
Manchu vs Hezhen	*ACE*	rs4343	G	9.455	0.0021
	*VDR*	rs7975232	C	5.427	0.0198
		rs11568820	C	19.535	9.88E-06
	*ACE*	rs4309	C	7.271	0.007
		rs4331	A	11.201	0.0008
		rs4341	G	10.228	0.0014
		rs4362	T	6.857	0.0088
	*IGF1*	rs1520220	C	4.419	0.0355
		rs972936	C	5.707	0.0169
	*AGT*	rs699	A	4.068	0.0437
		rs5050	G	5.551	0.0185
Mongolian vs Hezhen	*IL6*	rs2066992	T	11.107	0.0009
	*VDR*	rs7975232	C	11.175	0.0008
Mongolian vs Manchu	*IL6*	rs2066992	T	8.742	0.0031
	*VDR*	rs11568820	T	7.228	0.0072
	*IGF1*	rs5742714	G	4.243	0.0394

The average gene heterozygosity (AVE_HET) of the 23 SNPs in the five populations was 0.398 (Table 4). The average observed heterozygosity (OBS_HET) was 0.3957. The observed heterozygosity of rs1815739 and rs540874 in five populations was relatively large. The observed heterozygosity of rs4762 was the lowest. The F_ST_ values of the 23 SNPs among the five populations varied from 0.0009 to 0.0137, with an average of 0.0049, that is, 0.49% genetic variation existed between populations and 99.51% genetic variation existed within populations (Table 5). The gene flow of rs3856806 and rs1815739 was relatively large, and the mean value of Nm was 50.6913.

**TABLE 4:** summary of heterozygosity statistics for 23 SNPs.

Loci	Sample size	Obs_Hom	Obs_Het	Exp_Hom^a^	Exp_Het^a^	Nei^b^	Ave_Het
rs699	1748	0.6899	0.3101	0.6844	0.3156	0.3154	0.3144
rs4762	1728	0.8692	0.1308	0.8737	0.1263	0.1262	0.1187
rs5051	1712	0.6904	0.3096	0.6794	0.3206	0.3204	0.3231
rs5050	1728	0.735	0.265	0.7213	0.2787	0.2785	0.2729
rs3856806	1746	0.6999	0.3001	0.7122	0.2878	0.2876	0.2918
rs2066992	1764	0.5714	0.4286	0.5531	0.4469	0.4467	0.454
rs1617640	1760	0.592	0.408	0.5915	0.4085	0.4082	0.4166
rs551238	1764	0.576	0.424	0.5803	0.4197	0.4195	0.4246
rs1815739	1760	0.4773	0.5227	0.5045	0.4955	0.4952	0.4945
rs540874	1728	0.4884	0.5116	0.5044	0.4956	0.4953	0.4945
rs5742714	1752	0.7671	0.2329	0.7585	0.2415	0.2414	0.2305
rs1520220	1762	0.5289	0.4711	0.5271	0.4729	0.4727	0.4728
rs5742612	1764	0.6088	0.3912	0.6115	0.3885	0.3882	0.3897
rs972936	1762	0.5278	0.4722	0.5252	0.4748	0.4745	0.4741
rs7975232	1764	0.6088	0.3912	0.6159	0.3841	0.3839	0.3968
rs757343	1,620	0.7037	0.2963	0.7075	0.2925	0.2923	0.3042
rs2228570	1736	0.5472	0.4528	0.5191	0.4809	0.4806	0.4805
rs1156882	1728	0.5255	0.4745	0.5186	0.4814	0.4811	0.4733
rs4309	1760	0.5432	0.4568	0.5293	0.4707	0.4704	0.4671
rs4331	1764	0.5431	0.4569	0.5346	0.4654	0.4651	0.4619
rs4341	1762	0.5437	0.4563	0.5341	0.4659	0.4656	0.4624
rs4343	1762	0.5414	0.4586	0.5347	0.4653	0.465	0.4621
rs4362	1758	0.5199	0.4801	0.5238	0.4762	0.4759	0.4726
Mean	1745	0.6043	0.3957	0.6019	0.3981	0.3978	0.398
St. Dev		0.1005	0.1005	0.1003	0.1003	0.1002	0.1009

a Expected homozygosty and heterozygosity were computed using Levene (1949).

b Nei’s (1973) expected heterozygosit.

**TABLE 5:** Summary of the F-Statistics and gene flow for all the SNPs in five populations.

Loci	Sample size	Fis	Fit	Fst	Nm^a^
rs699	1748	0.0069	0.0123	0.0055	45.5111
rs4762	1728	−0.0257	-0.0207	0.0049	50.9974
rs5051	1712	0.0228	0.0279	0.0052	48.2872
rs5050	1728	0.0338	0.038	0.0043	57.7779
rs3856806	1746	−0.024	−0.0229	0.0011	233.5086
rs2066992	1764	0.04	0.0532	0.0137	17.9482
rs1617640	1760	−0.0027	0.0017	0.0044	56.6706
rs551238	1764	−0.0338	−0.0318	0.0019	130.0856
rs1815739	1760	−0.0617	−0.0607	0.0009	264.183
rs540874	1728	−0.0356	−0.0343	0.0013	195.3216
rs5742714	1752	0.0327	0.0365	0.0039	63.7296
rs1520220	1762	0.0164	0.019	0.0026	95.5691
rs5742612	1764	−0.0205	−0.0191	0.0014	178.7291
rs972936	1762	0.0147	0.0179	0.0032	76.7436
rs7975232	1764	−0.0614	−0.0486	0.0121	20.3944
rs757343	1620	−0.05	−0.0418	0.0078	31.9331
rs2228570	1736	0.0887	0.0939	0.0057	43.3971
rs11568820	1728	0.0301	0.0406	0.0108	22.8355
rs4309	1760	0.0405	0.0436	0.0033	75.9503
rs4331	1764	0.0286	0.0341	0.0056	44.638
rs4341	1762	0.0321	0.0371	0.0052	48.2127
rs4343	1762	0.0281	0.0328	0.0049	51.1232
rs4362	1758	0.006	0.0094	0.0034	72.6963
Mean	1745	0.0065	0.0114	0.0049	50.6913
St. Dev		0.0371	0.0376	0.0033	67.5013

a Nm = Gene flow estimated from F_ST_ = 0.25 (1 - F_ST_)/F_ST_.

### Haplotype Analysis

**FIGURE 2:**
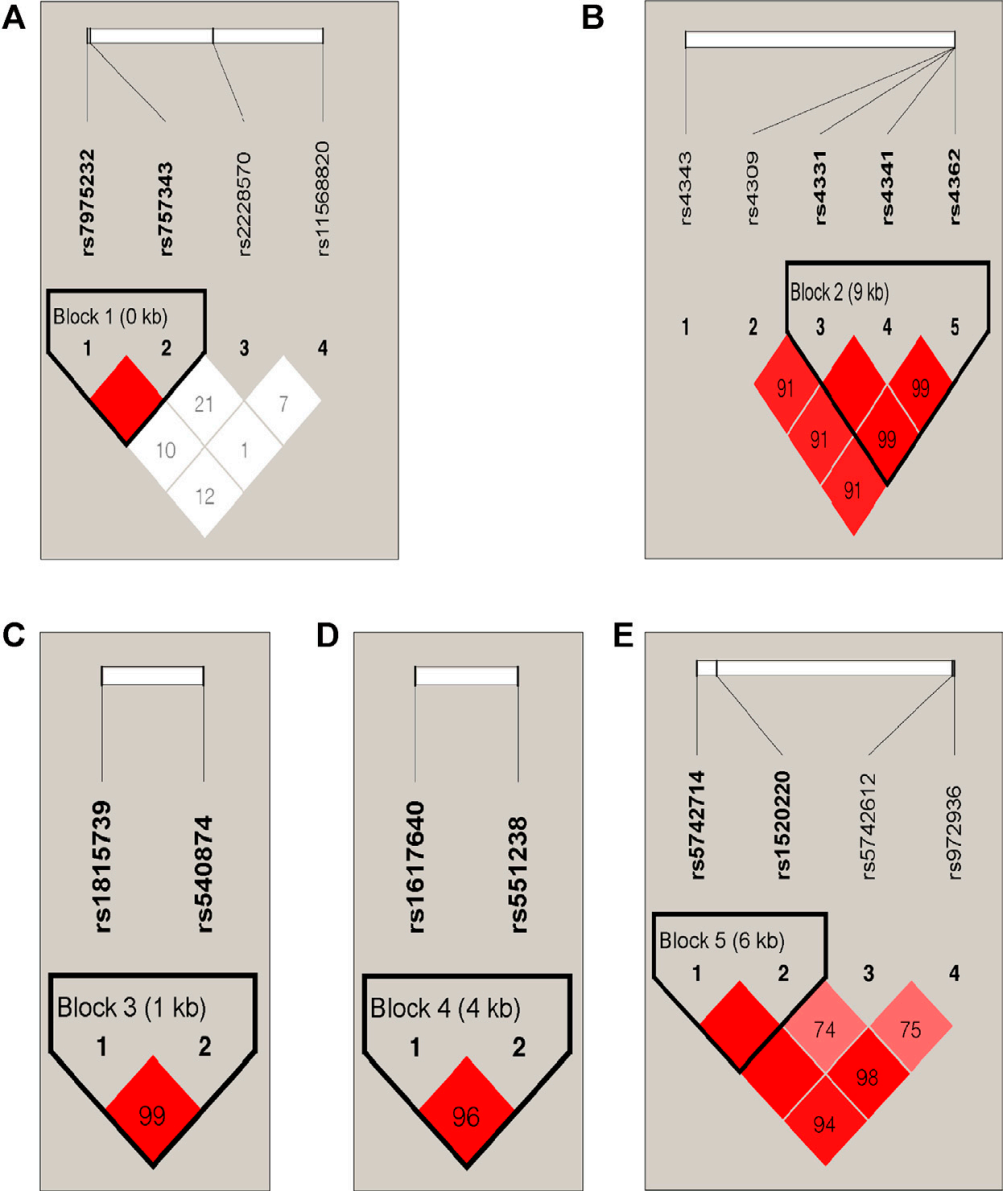
LD Plot. **(A)** The block1 containing two SNPs was constructed in the *VDR* gene. **(B)** The block2 containing three SNPs was constructed in the *ACE* gene. **(C)** The block3 containing two SNPs was constructed in the *ACTN3* gene. **(D)** The block4 containing two SNPs was constructed in the *EPO* gene. **(E)** The block5 containing two SNPs was constructed in the *IGF1* gene.

There were five blocks in 23 SNPs, the *r* ^2^ threshold of haplotype blocks were 0.8 Five blocks were distributed in *VDR*, *ACE*, *ACTN3*, *EPO* and *IGF1* genes (Table 6; Figure 2). The five blocks with statistical differences were mainly concentrated in *VDR* and *ACE* genes. The results showed that there were differences in haplotype distribution among the five ethnic groups. A block1 containing two SNPs was constructed in the *VDR* gene. The most common haplotype was CC, followed by AT and AC. The frequency distribution of CC was statistically significant between Daur and Ewenki (*P* = 0.0167) and between Daur and Mongolian (*P* = 0.0101). The frequency distribution of AT in Daur and Ewenki (*P* = 0.0045), Daur and Manchu (*P* = 1.00E-04), and Daur and Mongolian (*P* = 0.0071) were statistically significant. The frequency distribution of AC in Daur and Hezhen (*P* = 1.00E-04), Daur and Manchu (*P* = 0.0012), Ewenki and Manchu (*P* = 0.004) has statistical significance. The block2 containing three SNPs was constructed in the ACE gene. The most common haplotype was GCC, followed by AGT and GCT. The frequency distribution of AGT in Daur and Manchu (*P* = 0.0073), Ewenki and Manchu (*P* = 0.0475) is statistically significant, GCT in Daur and Hezhen (*P* = 0.0311), Daur and Manchu (*P* = 0.0013), Daur and Mongolian (*P* = 0.0339). Ewenki and Manchu (*P* = 0.0206) is statistically significant.

**TABLE 6:** Haplotype frequencies in five ethnic populations.

Gene	Block	Haplotype	Daur	Hezhen	Ewenki	Manchu	Mongolian
*VDR*	Block 1	CC	0.764^b^ ^,^ ^d^	0.788	0.664	0.723	0.67
		AT	0.134^b^ ^,^ ^c^ ^,^ ^d^	0.173	0.236	0.232	0.215
		AC	0.102^a^ ^,^ ^c^	0.039	0.100^c^	0.046	0.115
*ACE*	Block 2	GCC	0.605	0.554	0.609	0.638	0.613
		AGT	0.38^c^	0.398	0.383^c^	0.295	0.33
		GCT	0.013^a^ ^,^ ^c^ ^,^ ^d^	0.034	0.007^c^	0.05	0.038
*ACTN3*	Block 3	CG	0.571	0.522	0.541	0.548	0.557
		TA	0.425	0.476	0.452	0.452	0.443
*EPO*	Block 4	AT	0.691	0.72	0.644	0.704	0.66
		CG	0.281	0.258	0.322	0.279	0.302
		AG	0.026	0.02	0.014	0.014	0.019
*IGF1*	Block 5	CC	0.633	0.642	0.596	0.575	0.625
		CG	0.227	0.216	0.288	0.258	0.271
		GG	0.139	0.142	0.116	0.167	0.104

a Compared with Hezhen, p < 0.05.

b Compared with Ewenki, p < 0.05.

c Compared with Manchu, p < 0.05.

d Compared with Mongolian, p < 0.05.

### Inter Population Genetic Distances

**FIGURE 3:**
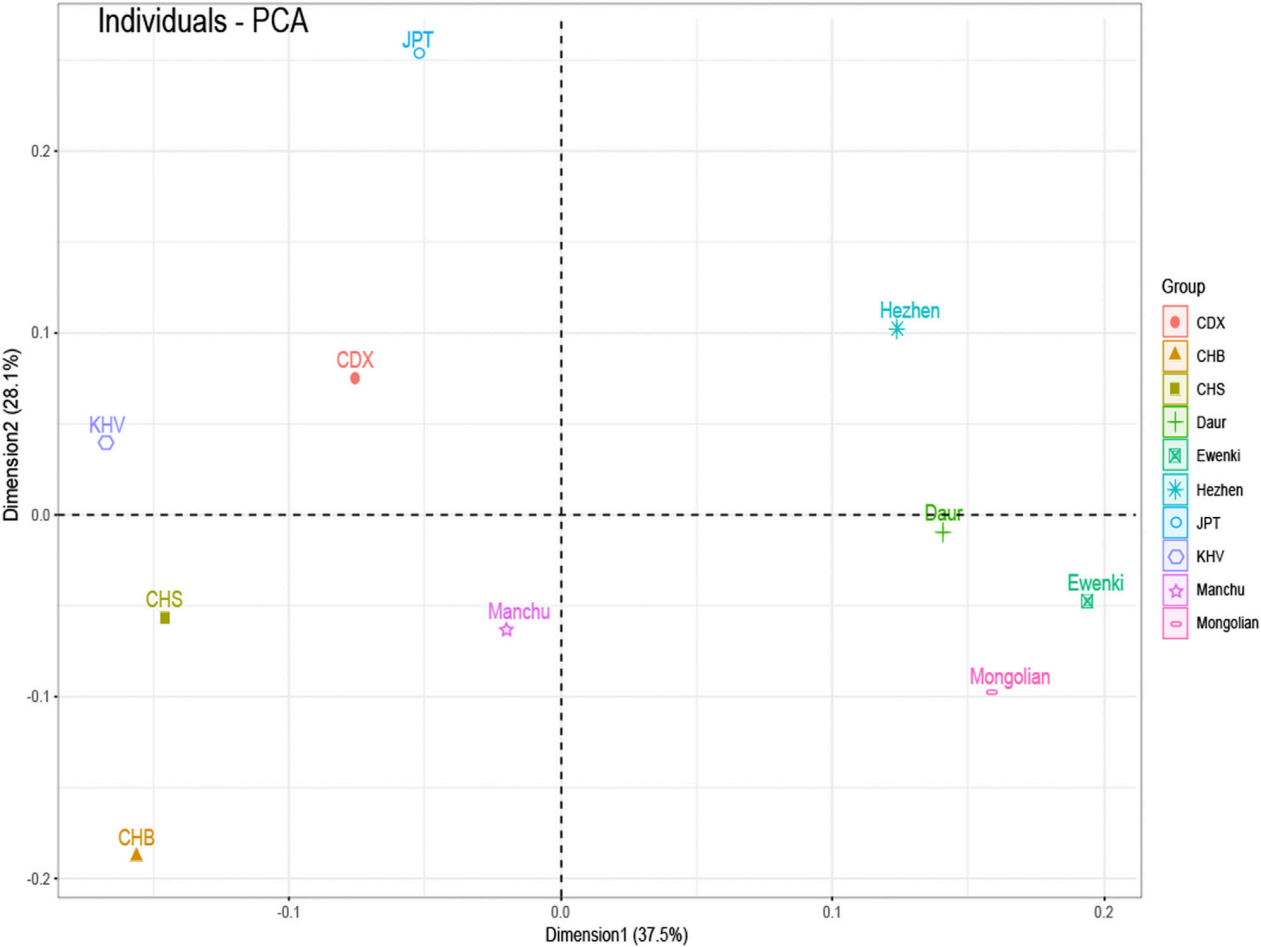
PCA based on the minimum allele frequency of the Asia populations. Note: CDX: Chinese Dai in Xishuangbanna; CHB: Han Chinese in Beijing; CHS: Southern Han Chinese; JPT: Japanese in Tokyo; KHV: Kinh in Ho Chi Minh City, Vietnam.

**FIGURE 4:**
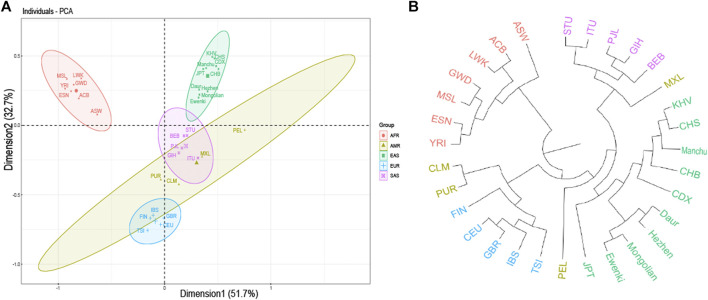
PCA and Neighbor-joining tree results. **(A)** PCA based on the minimum allele frequency of the world populations. **(B)** Neighbor-joining tree based on genetic distance from the world population genotype data. Note: CHB: Han Chinese in Beijing; JPT: Japanese in Tokyo; CHS: Southern Han Chinese; CDX: Chinese Dai in Xishuangbanna; KHV: Kinh in Ho Chi Minh City, Vietnam; CEU: Utah residents (CEPH) with Northern and Western European Ancestry; TSI: Toscani in Italia; FIN: Finnish in Finland; GBR: British in England and Scotland; IBS: Iberian Population in Spain; YRI: Yoruba in Ibadan, Nigeria; LWK: Luhya in Webuye, Kenya; GWD: Gambian in Western Divisions in the Gambia; MSL: Mende in Sierra Leone; ESN: Esan in Nigeria; ASW: Americans of African Ancestry in SW United States; ACB: African Caribbeans in Barbados; MXL: Mexican Ancestry from Los Angeles United States; PUR: Puerto ricans from Puerto rico; CLM: Colombians from Medellin, Colombia; PEL: Peruvians from Lima, Peru; GIH: Gujarati Indian from Houston, Texas; PJL: Punjabi from Lahore, Pakistan; BEB: Bengali from Bangladesh; STU: Sri Lankan Tamil from the United Kingdom; ITU: Indian Telugu from the United Kingdom.

F_ST_ value between five populations based on 23 SNPs indicated that the F_ST_ values of Daur and Mongolian (0.0026), Daur and Ewenki (0.0027), Mongolian and Ewenki (0.0006) are relatively small (Supplementary Table S2). According to the Nei’s genetic distance of the five ethnic groups based on UPGMA method (Supplementary Table S3). The genetic distance between Daur and Mongolian was relatively close (0.0035); the genetic distance between Mongolian and Ewenki was the closest (0.0007); The genetic distance between Daur and Ewenki was relatively close (0.0036). According to the PCA plot of the Asia populations (Figure 3), PC1 and PC2 accounted for 37.5 and 28.1% of the total genetic variation, respectively. The genetic distance between Daur, Ewenki and Mongolian were relatively close, which was consistent with the result of the FST value and the Nei’s Genetic Distance between the five ethnic groups (Supplementary TableS2,3). According to the PCA plot of the world populations (Figure 4A), PC1 and PC2 accounted for 51.7 and 32.7% of the total genetic variation, respectively. PCA plot divided the 31 world populations into five groups, namely AFR, AMR, EAS, EUR, and SAS, which named according to their geographic location of African, American, East Asian, European and South Asian. Population belonging to the same large group are generally clustered together, which are consistent with results from the phylogenetic tree of the world populations (Figure 4B). We found that the five ethnic groups included in the study were clustered in one cluster with the Asian population. In addition, the mean F_ST_ values and the mean Nm values of the 23 SNPs among the 31 populations was 0.098, 2.3017, respectively (Supplementary Table S4). According to the analysis of molecular variance (AMOVA) among the 31 populations, the percentage of variation among groups was 0.83%, while the percentage of variation within populations was 99.15% (Supplementary Table S5).

## Discussion

Different nations have formed specific genetic structures of different cultures, phenotypes and languages under the natural selection of different environments, material conditions and various pathogens. In East Asia, China has the largest Han population in the world, with 55 officially recognized ethnic groups making up their specific cultural backgrounds. They speak more than nine language families in China (Chen et al., 2019). Among them, five ethnic groups belonging to the Altaic language family in Heilongjiang province in northern China may have stronger physical fitness (Bi, 1993), the performance of the basic ability of human muscle activity. Some studies have found that there is a significant association between genotype and skeletal muscle phenotype. For example, the presence of SNPs is associated with better skeletal muscle strength performance (Khanal et al., 2020). We selected the 23 SNPs included in this study were all focused on genes related to skeletal strength, to further study the genetic composition and phylogeny of the five ethnic groups. 23 SNPs are consistent with the Hardy Weinberg equilibrium. In addition, in the pair comparison of the five populations studied, the genetic differences were mainly found on genes *IL6*, *VDR*, *AGT*, *ACE* and *IGF1*. for example, AGT encodes angiotensinogen, a protein involved in the renin-angiotensin-aldosterone system (RAAS) and is related to muscle growth (Ben-Zaken et al., 2019). *IGF1* is an important regulator not only of muscle mass and function, but also of bone. This is true not only during development, but throughout the human life cycle (Moriwaki et al., 2019). Vitamin D levels are closely related to the presence of vitamin D receptors in most human exoskeletal cells, and exposure to vitamin D in skeletal muscle leads to the expression of multiple myogenic transcription factors that promote the proliferation and differentiation of muscle cells (Wiciński et al., 2019). The angiotensin-converting enzyme (*ACE*) gene is associated with superior muscle metabolic performance and muscle endurance (Vaughan et al., 2013). Erythropoietin plays an important role in regulating metabolic homeostasis and bone remodeling (Suresh et al., 2019). Interleukin-6 (*IL-6*), the prototype of muscle factor, was identified as a muscle-derived cytokine 15 years ago (Karstoft and Pedersen, 2016). F_ST_ plays a core role in population and evolutionary genetics, it can reflect the degree of genetic differentiation between populations (Meirmans and Hedrick, 2011). The F_ST_ values of the 23 SNPs among the five ethnic groups varied from 0.0009 to 0.0137. There is almost no genetic differentiation in each population. According to the mean value of Nm, indicating that genetic differentiation did not occur between populations, but was mainly caused by genetic differentiation within populations, this is consistent with the population genetic differentiation results shown by the F_ST_ value of this study. We found that there was little difference in genetic distance between the five populations studied on the whole, this may because the five ethnic groups are all located in Heilongjiang province. which is consistent with the geographical location of the population (Tian et al., 2008). In addition, a total of five blocks exist in 23 SNPs (Figure 2). We concluded that rs7975232 and rs1815739 were statistically different in the five ethnic groups based on the F_ST_ values (Table 5). The same gene can perform different functions in the body, we found that the rs7975232 of *VDR* gene was related to the obesity and diabetes, it is also as the genetic makers of them. rs7975232 polymorphism of *VDR* gene was found to be positively correlated with obesity according to skin fold thickness and body fat rate in Chinese Han population (Shen et al., 2019). Another recent study also found that rs797523 polymorphism appears to be associated with overweight/obesity (Wang et al., 2021). The five ethnic groups in Heilongjiang province may be at higher risk of obesity or overweight due to environmental、eating habits and genetic factors because they are located in the extremely cold area of northern China. As we known, obesity is an important risk factor for diabetes. Meanwhile, it may reveal that the ethnic groups in the extremely cold area of northern China may be susceptible to diabetes to a certain extent (Li et al., 2020). Interestingly, another locus of significant genetic variation explains exactly how extreme cold affects skeletal muscle in humans. The positive selection of the allele of rs1815739 in cold climates provides the mechanism, that is, the slower type of I MyHC in the α-actinin-3 muscle, combined with a shift in neuronal muscle activation to increase muscle tone rather than obvious tremor, supporting the key thermogenesis of human skeletal muscle during cold exposure (Wyckelsma et al., 2021). Therefore, we believe that the genetic difference of rs1815739 and rs7975232 among the five ethnic groups may be caused by the fact that the five ethnic groups are located in Heilongjiang province, a high-latitude and severe cold region in China. The largest component of genotypic variation is the reduction of high-order data (all genotypes) to low-order variation. According to the PCA results of the Asian population, the genetic distances of Daur, Ewenki, and Mongolian were relatively close, indicates that in the mixing process of history and modern times. There may be gene exchange between Daur and Mongolian and Ewenki to some extent (Liu et al., 2007; Gao et al., 2006), which was consistent with the result of the Nei’s genetic distance and F_ST_ values between the five ethnic groups. There are also studies showing that from the perspective of linguistic kinship, immigration history and origin, the kinship between the Mongolian and the Daur is very close, which indicates that the two groups in each pair may be of the same origin (Hou et al., 2007). According to the world population phylogeny tree and PCA, the genetic distance between the five populations and the Asian population is relatively close, and they are clustered with the Asian population. The genetic variation of 31 populations occurred mainly within the population (Supplementary Table S4,5).

In Conclusion, geographical and linguistic divisions have shaped the genetic structure of modern populations. Cluster analysis shows that the five ethnic groups in Heilongjiang province are clustered together with the East Asian ethnic groups. The genetic distance between Daur, Mongolian and Ewenki is closer, in order to better study the genetic characteristics of skeletal muscle strength related genes in different population, in addition to the more national, cultural, geographical and linguistic diversity group, also need more genome data combining with archaeological data and population history for further analysis and validation.

## Data Availability

The original contributions presented in the study are included in the article/Supplementary Material, further inquiries can be directed to the corresponding author.
